# Cytoreductive Nephrectomy Promoted Abscopal Effect of Camrelizumab Combined With Radiotherapy for Metastatic Renal Cell Carcinoma: A Case Report and Review of the Literature

**DOI:** 10.3389/fimmu.2021.646085

**Published:** 2021-06-15

**Authors:** Min Wu, Jie Liu, Samuel Seery, Xue Meng, Jinbo Yue

**Affiliations:** ^1^ Cheeloo College of Medicine, Shandong University, Jinan, China; ^2^ Department of Radiation Oncology, Shandong Cancer Hospital and Institute, Shandong First Medical University and Shandong Academy of Medical Sciences, Jinan, China; ^3^ Health Research, Faculty of Health and Medicine, Lancaster University, City of Lancaster, United Kingdom

**Keywords:** clear cell renal cell carcinoma, camrelizumab, abscopal effect, pseudoprogression, MLR

## Abstract

There is little evidence around Camrelizumab combined with cytoreductive nephrectomy (CN) and radiotherapy (RT) as a treatment option for metastatic renal cell carcinoma (mRCC). The influence of CN on immune responses and the abscopal effect are not well understood. In this paper, we report a case of anti-programmed cell death-1 (PD-1) treated with combined RT once CN reduced the primary tumor burden (TB). This patient also encountered an increased response to targeted radiotherapy after immune resistance. We also observed a macrophage-to-lymphocyte ratio (MLR) peak, which may be correlated with subsequent pseudoprogression after thoracic radiotherapy. Consequently, even with the disease, this patient has remained stable. This peculiar instance suggests there is a need to investigate the underlying mechanisms of CN in promoting the abscopal effect during immunotherapy when combined with RT. It also suggests that there is a need for further investigation into the role of RT in overcoming immune resistance, and the value of MLR in predicting pseudoprogression. We hypothesize that a heavy tumor burden might suppress the abscopal effect, thereby ensuring that CN promotes it. However, radiotherapy may overcome immune resistance during oligoprogression.

## Introduction

Renal cell carcinoma (RCC) accounts for approximately 85% of all pathological renal carcinomas. Among all of these cases, 70% are clear cell renal cell carcinomas (ccRCC) (1). Approximately 30% of all kidney carcinoma patients have multiple distant metastases such as lung, bone, brain, and other organs upon diagnosis, of which the 5-year survival rate is only around 8% (2). Renal carcinoma has strong immunogenicity. Cytokine therapy was generally offered before the discovery of a number of targeted therapies, which eventually became standard first-line interventions for ccRCC (3). However, immunotherapies have now become the first-line therapy and have gradually become a new treatment mode for metastatic, inoperable, medium-high risk ccRCC (4–6).

Based on the results of various clinical trials, immune monotherapy combined with CN or RT appear to prolong survival (7, 8). CN therefore appears to reduce the tumor burden so that the immunotherapeutic effect is enhanced. Immunotherapy combined with RT also improves survival, although the abscopal effect is infrequent (9–11). The abscopal effect is a rare phenomenon associated with radiotherapy (11). It refers to the release of immunocompetent molecules from apoptosing tumor cells after local radiotherapy and the stimulation of immune responses, which leads to the reduction and control of tumor lesions without RT (12). Multiple studies have revealed that combined radiotherapy and immunotherapy can be more effective at overcoming tumor immunosuppression and increasing the incidence of abscopal effect compared to RT alone, regardless of the type of immune checkpoint inhibitor combined with either CTLA-4 or PD-1/PD-L1 inhibitors (13, 14).

However, there is no data on the effect of CN in immunotherapeutics when combined with RT or the potential abscopal effect that comes with it. This is the first report of a patient who was administered Camrelizumab combined with CN and radiotherapy as a second-line treatment for mRCC. In this instance, CN appeared to promote the abscopal effect when receiving immunotherapy and RT. RT plays an important role in oligoprogressive metastasis and may be useful in overcoming immune resistance.

## Case Description

**Figure 1:**
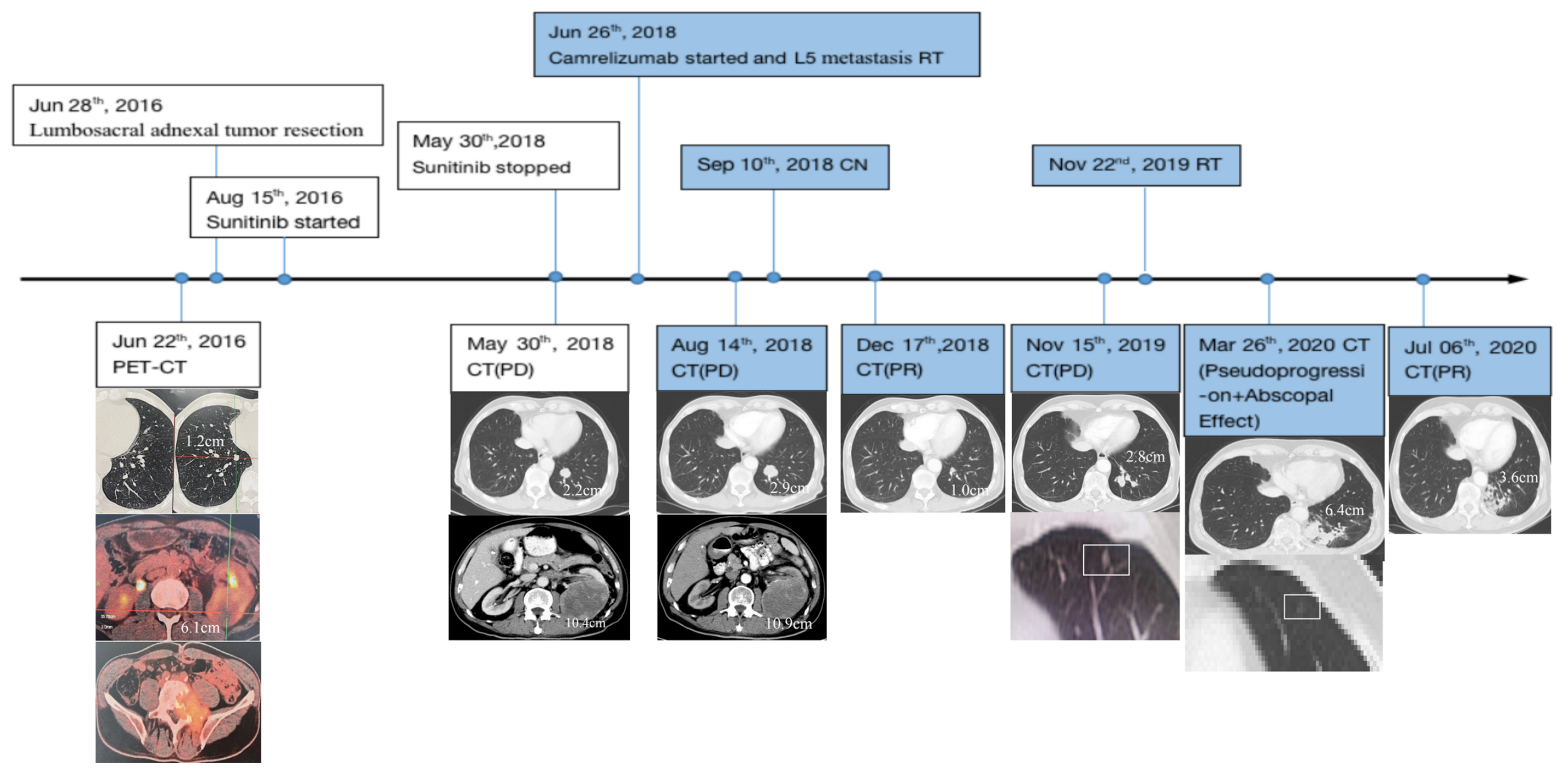
Disease status timeline and PET/CT scan of primary and metastatic lesions accompanied by treatment regimens. CN, cytoreductive nephrectomy; RT, radiotherapy; PET/CT, positron emission tomography-computed tomography/computed tomography; PD, progressive disease; PR, partial response; Point-in-time filled with color represents continuous use of Camrelizumab.

A 65-year-old man presented at an outpatient clinic with a chief complaint of excruciating pain around his waist and lower left limb for more than 10 months. Physical examination revealed a left lumbar mass, which warranted further examination. On June 22, 2016, positron emission tomography-computed tomography (PET/CT) enabled clinicians to identify a 6.1 cm mass located in the left kidney with multiple bone and double lung metastases. The largest lesion was approximately 1.2 cm and was located in the inferior lobe of the right lung. Lumbar magnetic resonance imaging (MRI) highlighted the existence of a soft mass near the fifth lumbar vertebra (L5) (**Figure 1**).

To relieve the patient’s pain, lumbosacral adnexal tumor resection from the fourth lumbar vertebra to the first sacral vertebra with internal fixation and pyramidal plasty of the first sacral vertebra was performed on June 28, 2016. Post-operative pathology determined metastatic ccRCC. Tissue-based genetic testing also showed high VEGFR1 mRNA expression; consequently, treatment with Sunitinib was initiated in mid-August 2016. However, serum creatinine level rose from a pre-Sunitinib level of 74 μmol/L to a high of 202 μmol/L over approximately two years. Thyroid function appeared normal prior to receiving the targeted therapy, but thyroid stimulating hormone levels also progressively rose to 85.23 mIU/L while the T4 thyroid hormone decreased to 7.10 pmol/L until Sunitinib was discontinued.

**Figure 2:**
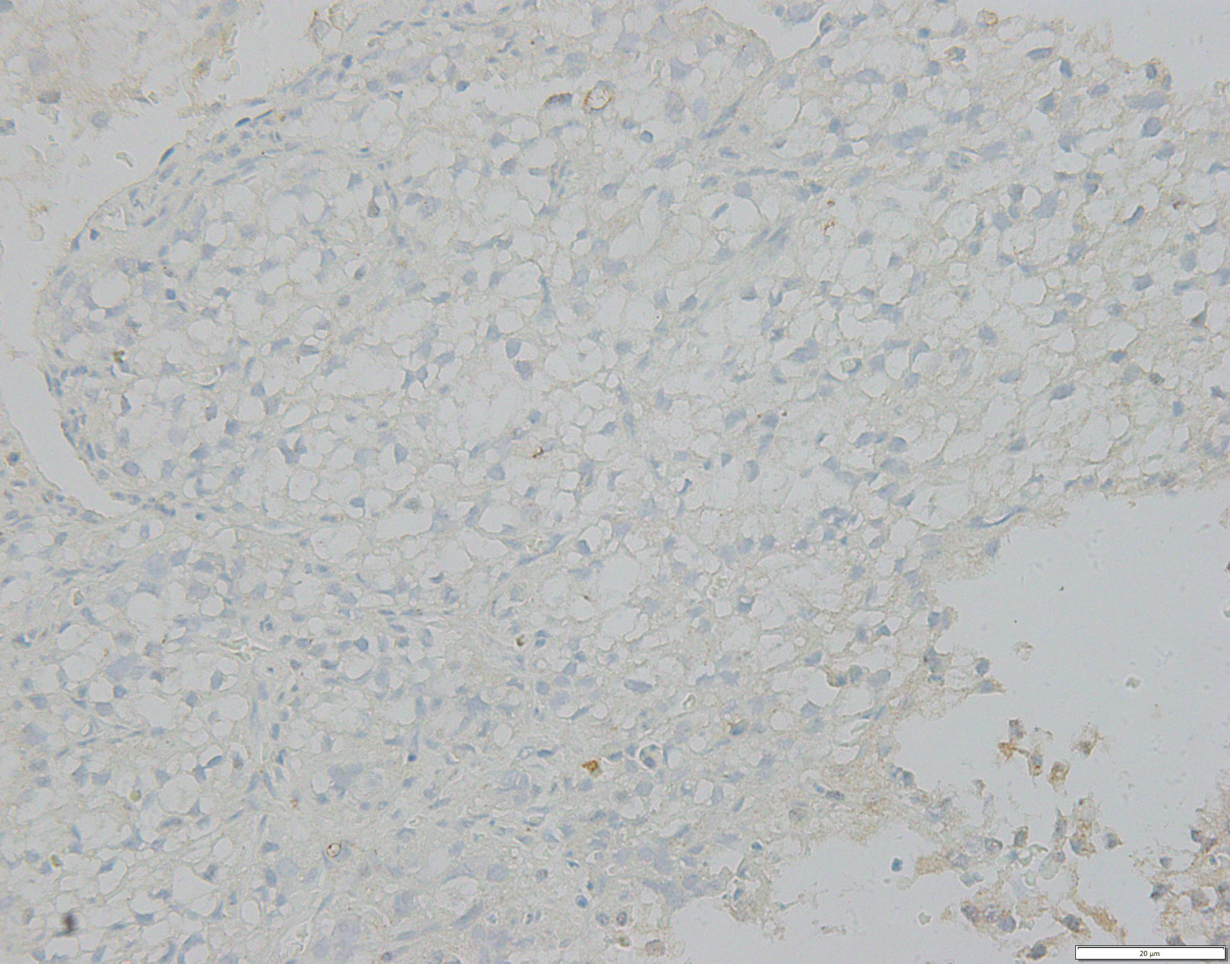
FFPE tissue photomicrograph from metastatic lesion stained for negative PD-L1 assessment. PD-L1, programmed cell death receptor-ligand 1.

On May 30, 2018, when the patient first underwent a routine examination in our hospital, CT scans revealed that the left renal mass had enlarged to 10.4 cm with the largest 2.2 cm lesion transforming into the left lung, and vertebral metastases had recurred. These findings met the standard definition for progressive disease (PD) according to RECIST guidelines (15). Programmed cell death receptor ligand 1 (PD-L1) expression tests were negative (**Figure 2**). We recommended a combined dose of axitinib and pembrolizumab as a second-line treatment. However, this patient declined and preferred a single dose of Camrelizumab (AiRuiKa™) instead, a programmed cell death 1 (PD-1) inhibitor at 200 mg q2W for four cycles in step with helical tomotherapy at 45 Gy in 15 fractions for the L5 metastasis lesion.

On August 14, 2018, CT re-examination showed that the disease had progressed globally, with the left renal primary lesion enlarging once more from 10.4 cm to 10.9 cm. The left pulmonary metastasis had also increased from 2.2 cm to 2.9 cm, with new metastases appearing in both lungs. It was suspected that this was due to heavy renal tumor burden; therefore, this patient underwent CN to the left renal mass on September 10, 2018. Post-operative pathological diagnosis confirmed ccRCC. The patient continued receiving 200 mg q3W Camrelizumab after CN.

On December 17, 2018, CT revealed that the size of the largest metastasis in the left lung was reduced to 1.0 cm after two post-operative cycles of Camrelizumab, which suggested a substantial disease response. This first abscopal effect manifesting through combined radiotherapy and immunotherapy occurred after CN. Subsequent re-examination reflected relative disease stability with regular immunotherapy; however, the lesion in the left inferior lobar bronchus increased to 2.8 cm even after 20 post-operative cycles of Camrelizumab, indicating disease progression according to the imRECIST standard (16).

On November 22, 2019, oligoprogressive lesion-targeted radiotherapy was commenced at 45 Gy in 15 fractions with an MLR peak during radiation (**Figure 2**). RT was completed on December 12, 2019 without severe adverse reactions or discontinuation of Camrelizumab. Unfortunately, CT scans taken on the March 16, 2020 showed that the targeted metastatic lesion continued to grow to 6.4 cm even after radiation. Interestingly, the density of another small metastatic nodule theoretically outside the field of radiation was lower than previously recorded.

According to the imRECIST standard (16), we recommended a tissue biopsy to determine whether there was pseudoprogression of the disease. However, the patient declined. The patient continued Camrelizumab treatment, resulting in a second abscopal effect which showed that the superior lobe of left lung metastasis had shrunk (**Figure 1**). After seven further cycles of Camrelizumab, CT revealed that the lesion in the left inferior lobe had also shrunk in size from 6.4 cm to 3.6 cm, which appeared to confirm suspected pseudoprogression. Until now, the patient’s disease has remained stable with Camrelizumab maintenance. The efficacy of Camrelizumab in this patient was unaffected by negative PD-1/PD-L1 expression, and all adverse reactions caused by Sunitinib diminished without additional severe immune-related adverse events (irAEs) except for minor renal function damage.

The patient also had normal fasting blood glucose levels from November 2018 to August 2019, which is peculiar for a man with a history of diabetes for more than ten years without discontinuing insulin. Hematological indices, including white blood cell (WBC), neutrophil (N), and lymphocyte (L) levels, remained stable throughout the treatment. However, the number of monocytes and the macrophage-to-lymphocyte ratio (MLR) peaked during radiotherapy for lung metastasis, which may be correlated with subsequent pseudoprogression (**Figures 1** and **4**).

## Discussion

As the most common pathological type of renal carcinoma, ccRCC is associated with a dismal prognosis. Furthermore, the median PFS of mRCC ranges from six to ten months (1). Current evidence suggests that ccRCC is a highly immunogenic tumor and should therefore be subjected to immune checkpoint inhibitor-based interventions (4, 6). Our report highlights the remarkable effect of Camrelizumab when combined with CN and radiotherapy regardless of PD-L1 expression. Abscopal effects associated with RT combined with immunotherapy occurred twice during treatment after CN. Local radiotherapy for oligoprogressive lesions was also beneficial for immune resistance and pseudoprogression and was probably associated with the MLR peak in blood.

According to various animal models, the immune microenvironment changes after hypofractionated RT because PD-L1 expression is upregulated in tumors, weakening the immunosuppressive effect and activating cytotoxic T cells; this in turn reduces tumor invasive myeloid-derived suppressor cells (MDSCs) (17). In two previous retrospective studies, researchers have found that the overall survival of melanoma patients with signs of the abscopal effect after radiotherapy combined with CTLA-4 inhibitors was significantly longer than for those without (12, 18). Some studies have also reported an abscopal effect in mRCC during radiation with checkpoint inhibitor immunotherapies such as nivolumab or pembrolizumab (19–21).

Our patient encountered abscopal effects twice, which may be related to the aforementioned mechanisms involved in the immune microenvironment. He was treated with Camrelizumab after Sunitinib resistance, which is the first report focusing on a Chinese PD-1 monoclonal antibody for ccRCC in addition to tirelizumab (22). However, after four cycles of Camrelizumab immunotherapy and irradiation, the disease continued to progress slowly. We then supplemented the strategy with CN; unexpectedly, the efficiency evaluation measure highlighted a partial response and the first abscopal effect occurred. We infer that CN reduced the number of tumor cells so that there were sufficient PD-1 proteins combined with PD-L1. This in turn activated T cells, enabling them to avoid being suppressed. Physiological memory accumulated over the previous RT six months, and there was a transformation in the remaining M1 macrophages in distant metastases which may lead to the first abscopal effect.

Cytoreductive nephrectomy always plays an unpredictable role for patients with mRCC (23). There are both advantages and futilities associated with this intervention (24). As immune checkpoint inhibitors are rapidly becoming the standard treatment for mRCC, the synergistic effect of CN combined with ICIs has been questioned once more. A previous retrospective study suggested that the median overall survival in the CN plus IO group was not attained, while the median OS in the IO arm was 11.6 months with an HR of 0.23 (7). Other similar studies exploring CN in the treatment with immunotherapy are yet to be initiated; therefore, the evidence base is relatively immature. Unfortunately, there are still no relevant studies that report CN promoting the abscopal effect. Our patient benefited from CN combined with immunotherapy and radiotherapy because of the accelerated abscopal effect experienced with this treatment. Although RT is known to stimulate immunotherapy and reduce tumor burden as well, the disease still progressed after RT for L5 metastasis in advance. However, after CN the disease response was PR immediately. It does have the possibility that CN reduced tumor burden with the accumulative effect of RT for L5. But we couldn’t evaluate the effect of CN and L5 radiotherapy on reducing the number of tumor cells respectively because we hadn’t detected TB timely. However, Patients with heavy tumor burden may be more capable of coping with CN, immunotherapy, and RT combinations, thereby providing a window of opportunity; however, further research is required.

Several previous studies have found that the level of CRP negatively correlates with both OS and cancer-specific survival (25–28). Reichle et al. suspected that tumor cells produce CRP, which is thought to be the reason why patients with larger tumor volumes have higher CRP levels (29). Tatokoro et al. also hypothesized that CRP could be used to accurately reflect tumor burden as a biomarker (30). In this case, the patient’s serum CRP level significantly reduced from 36 mg/L to 2.69 mg/L since Camrelizumab and RT for L5 metastasis intervention, as well as CN afterwards, which may reflect the combination therapy of ICI and RT does reduce the tumor burden. Another study found that tumor burden is associated with baseline T‐cell receptor β‐chain (TCRB) diversity, and that high diversity of TCRB increases the accumulation of CD4+ and CD8+ T cells. This in turn initiates further immune response and increases the likelihood of an improved prognosis (31). During radiotherapy, TCRB was enriched with tumor-associated antigens that enhanced the immune efficiency. RT indirectly activates T cells that infiltrate tumor lesions and increases TCRB diversity, which accelerates the efficacy of immune checkpoint inhibitors. Therefore, the combination of RT and immunotherapy can greatly enhance the anti-tumor effect.

This patient developed an acquired immune resistance after receiving Camrelizumab for more than one year. As a matter of course, we commenced RT even if the targeted lesion continued to grow, while the density of another small metastatic nodule outside the field of radiation lessened. After continuous immunotherapy, CT revealed that the radiated lesion decreased in size, which confirmed pseudoprogression and the abscopal effect. Immunotherapy resistance against PD-1/PD-L1 has been observed in different solid tumors (32). Patients with low expression of PD-L1 or TMB are more likely to acquire adaptive resistance to ICI. If OP refers to no more than two progressive metastases, more than 56% of the progression in solid tumors is oligoprogression in acquired resistance during ICI, which can easily be addressed with local RT (33). There is one previous report of a woman with metastatic anorectal mucosal melanoma who received CR after radiotherapy before encountering oligoprogression during nivolumab immunotherapy (34). Therefore, RT reversing acquired immune resistance is substantial.

A prospective study by Welsh et al. found that adjuvant radiation therapy can overcome resistance to anti-PD-1 treatment by inducing IFN-β production and elevating MHC class I expression (35). They also found that RT combined with OXPHOS inhibition not only reverses PD-1 resistance in non-small-cell lung cancer, but also enhances anti-tumor immunity (36). In 2019, researchers discovered that resistance to PD-1 checkpoint blockade may be overcome by ADAR1 loss (32). However, another study found that resistance to PD-1 blockade is associated with an inactivation of antigen presentation (37). Our patient had a negative PD-L1 expression and benefited from a second course of radiotherapy when he incurred Camrelizumab resistance, suggesting that there was an underlying acquired immune resistance. RT clearly reversed resistance to Camrelizumab, after which pseudoprogression and a second abscopal effect were observed. This peculiar occurrence presents several unknowns, and the role of RT in overcoming immune resistance requires further investigation.

CT imaging suggested that pseudoprogression manifests with temporary tumor enlargement or new lesions, which can actually be an indicator for improved prognosis (38). The overall incidence of pseudoprogression is approximately 6% in solid tumors and between 5.7 to 8.8% in renal carcinoma (9, 10). Radiotherapy changes the tumor microenvironment and enhances anti-tumor responses by inducing T lymphocyte aggregation in lesions (39). Another new pattern of progression within eight weeks of initial treatment of immunotherapy is called hyperprogression, which has several different definitions (40–42). The incidence of hyperprogression in lung cancer ranges from 5% to 19.2% (43) and generally predicts poor prognosis (44). Patients have a higher possibility of incurring hyperprogression when they are older (70+ years), have EGFR/ALK/MDM2/MDM4 mutations, or have multiple metastases (42, 44, 45). The factors with which we differentiate pseudoprogression and hyperprogression include patients’ clinical symptoms, serum levels of IL-8, ctDNA, and PET-CT (46).

**Figure 3:**
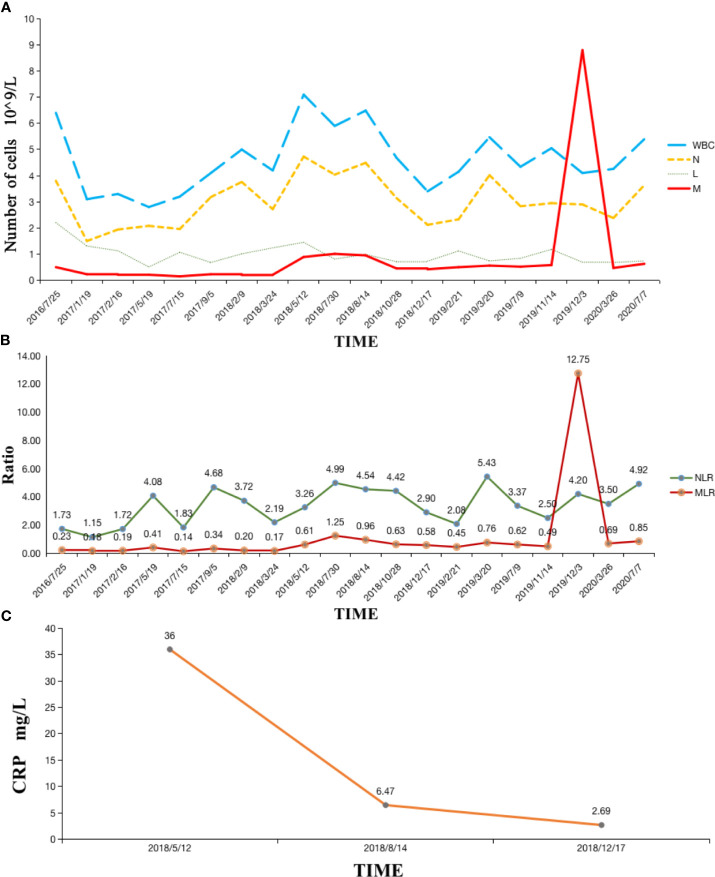
**(A)** Hematological indexes remained stable except the number of monocytes peaked during radiotherapy; **(B)** MLR remained stable and then peaked during radiotherapy; **(C)** Serum CRP level significantly reduced after Camrelizumab and RT. WBC, white blood cell; N, neutrophil; L, lymphocyte; M, macrophage; MLR, macrophage-to-lymphocyte ratio; NLR, neutrophil-to-lymphocyte ratio; CRP, C-reactive protein.

We observed a peak MLR in our patient’s regular blood work during radiotherapy, in which the number of monocytes was ten times greater than normal (**Figure 3**). In recent years, the role of macrophages in tumor immunotherapy has become controversial. We know that there are various sources of macrophages within different organs, while tumor-related macrophages (TAMs) are often considered to originate from circulatory monocytes (47). This constitutes what is known as an immunosuppressive triangle with Treg cells and MDSCs within the tumor microenvironment (48). TAMs can be divided into two distinct phenotypes according to different polarizations. Dormant M0-TAMs can be driven toward classical (M1) or alternative (M2) activation under the stimulation of the microenvironment (49). M1 type macrophages enhance immune responses and play an important anti-tumor role *via* pro-inflammatory cytokines, inducible nitric oxide synthase 2, and MHC class II molecules (50). M2 type macrophages are enriched in the hypoxic region of tumor tissues, expressing functions such as promoting angiogenesis, remodeling matrices, and suppressing adaptive immunity. The number of M2 type macrophages positively correlates with the progression of tumors, with many studies showing that TAMs predominantly display an M2-like phenotype (51).

**Figure 4:**
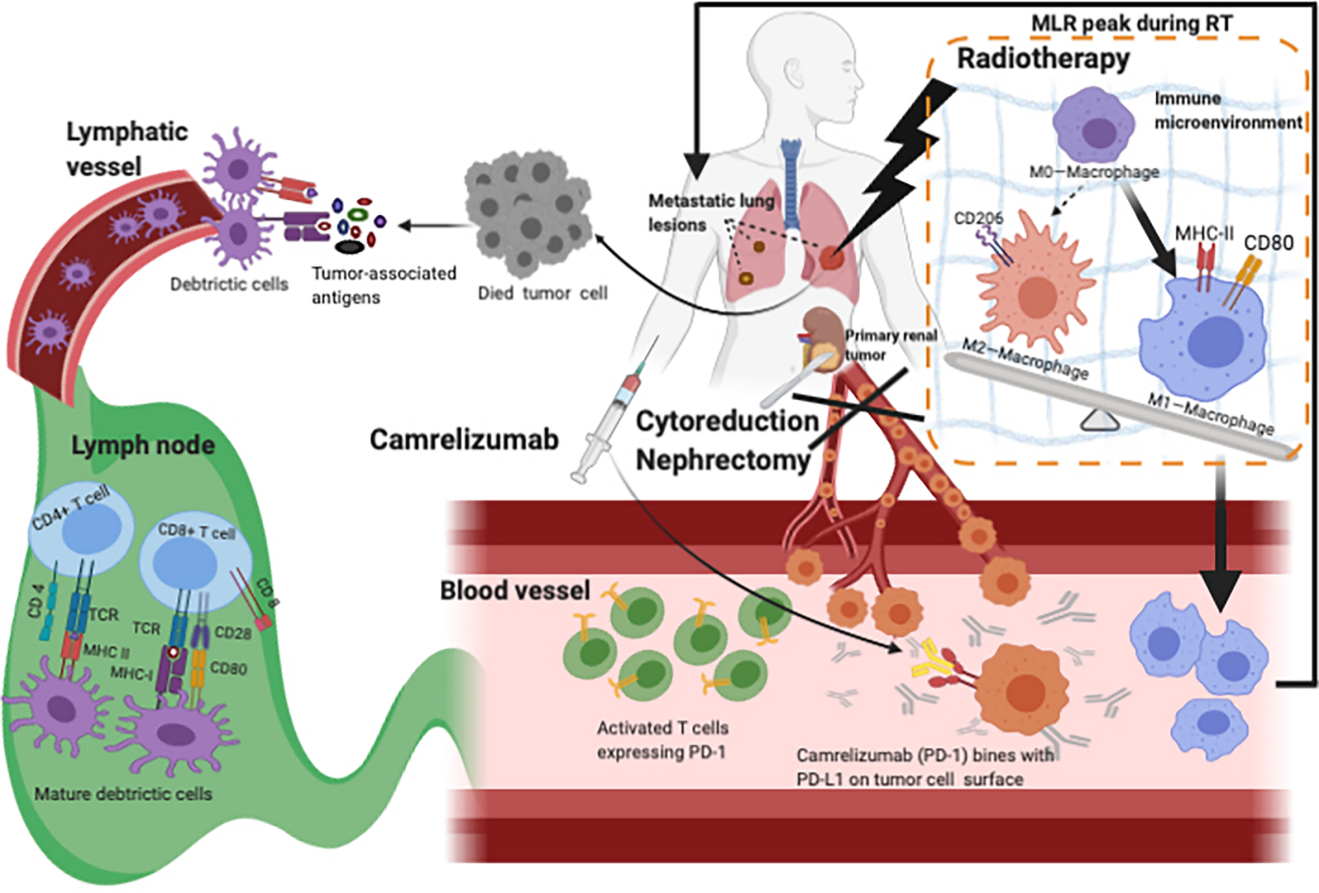
Hypothesis for the mechanism of Camrelizumab combined with CN and RT. RT destroys tumor cells so that more tumor-associated antigens can be released and recognized by tissue-resident DCs, accelerating the activation of T cells which increases the rate of activated T cells. With CN decreasing the TB, enough Camrelizumab could combine with PD-L1 expressed on tumor cells, so that T cells avoid being suppressed and work. During Camrelizumab prescription, radiation may change the microenvironment which might convert more M0-macrophages into M1-macrophages. More activated T cells and M1-macrophages wandered in blood vessels, which can account for pseudoprogression and abscopal effect. CN, cytoreductive nephrectomy; RT, radiotherapy; DCs, dendritic cells; TB, tumor burden; PD-L1, programmed cell death receptor-ligand 1.

For surface biomarkers however, CD80 and MHC II expression in M1 type macrophages were particularly high. Although this is not the case for CD206, which appears similar to M0 macrophages; the level of CD206 expression in M2 type macrophages is considerably higher. Under continuous immune microenvironment stimulation, low or intermediate radiation doses can influence changes in macrophage phenotype by the expression of surface molecular markers (52). An original study revealed that low-dose irradiation can program macrophages into an M1 phenotype, which leads to the normalization of tumor vasculature and aggregation of immune cells in tumor sites (53). Our patient probably underwent more M1 type macrophage transformation after RT. Then, with a residual memory of RT, tumor cells decreased during CN, and extra M1 macrophages had the opportunity to gather distant metastases through blood vessels, providing a possible reason for the MLR peak and pseudoprogression after radiotherapy (**Figures 1** and **4**).

To the best of our knowledge, this is the first reported case in which a patient apparently encountered temporary diabetes remission while receiving immunotherapy. All previous studies have found that type I diabetes is a common temporary side effect of immune checkpoint inhibitors (54, 55). Anti-PD-1/PD-L1 monoclonal antibody can induce insulin-dependent diabetes as one severe side effect (56, 57). A basic study found that low PD-L1 expression is associated with type I diabetes, where diabetes was reverted by upregulating PD-L1 expression to inhibit autoimmune response (58). Therefore, our case is peculiar and unprecedented, providing the first clinical evidence of this phenomenon. As such, we ought to consider the mechanisms that may be involved, specifically PD-L1 expression during treatment.

In conclusion, we observed a remarkable effect of CN combined with Camrelizumab and radiotherapy, regardless of PD-L1 expression. Interestingly, the abscopal effect associated with RT and immunotherapy occurred twice during treatment. CN also reduced the tumor burden, which may have promoted the first abscopal effect during RT. Local radiotherapy was again added when the patient encountered immune resistance, after which pseudoprogression and the second abscopal effect were observed. MLR during RT may also be a useful biomarker for predicting radiotherapeutic efficacy and prognosis. This is the first clinical evidence of this nature; therefore, we ought to consider the molecular mechanisms involved in radiation-related pseudoprogression, abscopal effect, and immune resistance. It is therefore of paramount importance that clinicians working in this field take the time to report any instances that may provide further insight.

## Data Availability Statement

The original contributions presented in the study are included in the article/supplementary material. Further inquiries can be directed to the corresponding authors.

## Ethics Statement

Written informed consent was obtained from the individual(s) for the publication of any potentially identifiable images or data included in this article.

## Author Contributions

MW, JL, SS, XM, and JY contributed to the study. JY designed the manuscript and approved the final manuscript. XM made critical appraisal. JL is responsible for providing patient information and overall framework arrangements. SS modified and edited this report. MW collected the CT images, analyzed data, and drafted the article. All authors contributed to the article and approved the submitted version.

## Funding

This work was supported by the following grants: National Natural Science Foundation of China (Grant No. 81871895),Young Taishan Scholars and Academic Promotion Program of Shandong First Medical University (Grant No. 2019RC003).

## Conflict of Interest

The authors declare that the research was conducted in the absence of any commercial or financial relationships that could be construed as a potential conflict of interest.
